# Ultrasensitive quantification of HIV-1 cell-to-cell transmission in primary human CD4^+^ T cells measures viral sensitivity to broadly neutralizing antibodies

**DOI:** 10.1128/mbio.02428-23

**Published:** 2023-12-08

**Authors:** Dmitriy Mazurov, Alon Herschhorn

**Affiliations:** 1 Division of Infectious Diseases and International Medicine, Department of Medicine, University of Minnesota, Minneapolis, Minnesota, USA; 2 Institute for Molecular Virology, University of Minnesota, Minneapolis, Minnesota, USA; 3 Institute for Engineering in Medicine, Center for Genome Engineering, University of Minnesota, Minneapolis, Minnesota, USA; 4 Microbiology, Immunology, and Cancer Biology Graduate Program, University of Minnesota, Minneapolis, Minnesota, USA; 5 The College of Veterinary Medicine Graduate Program, University of Minnesota, Minneapolis, Minnesota, USA; 6 Molecular Pharmacology and Therapeutics Graduate Program, University of Minnesota, Minneapolis, Minnesota, USA; Columbia University Medical College, New York, USA

**Keywords:** HIV-1, cell-cell transmission, broadly neutralizing antibodies

## Abstract

HIV-1 can efficiently replicate by direct transmission from infected to uninfected CD4^+^ T cells at confined local sites designated virological synapses (VSs). VSs are formed by cell junctions between HIV-1 envelope glycoproteins (Envs) on an infected cell and CD4 on an uninfected cell. These sites facilitate highly efficient viral transmission and contribute to HIV-1 evasion from neutralizing antibodies, but accurate quantification of the efficiency of cell-cell transmission is still challenging. Here, we developed an ultrasensitive HIV-1 cell-to-cell transmission assay that triggers the expression of the *nanoluciferase (nluc*) gene in target cells upon transmission and after reverse transcription of the HIV-1 RNA genome. The assay is based on the insertion of the *nluc* gene in an antisense orientation into the HIV-1 provirus. Nluc expression is blocked in virus-producing cells because the *nluc* gene contains an intron that can be efficiently spliced out only when mRNA is transcribed from the opposite (sense) strand. Thus, only sense mRNA that is spliced and subsequently reverse transcribed during transmission to target cells will support Nluc expression. Assay optimization resulted in a very low background, >99% splicing efficiency, high sensitivity, and wide dynamic range for the detection of cell-cell transmission in T cell lines and primary CD4^+^ T cells. The optimized reporter vector detects cell-cell transmission using single-round viral vectors and HIV-1 molecular clones, which provide viral proteins of different HIV-1 strains, and reproducibly measures the sensitivity of HIV-1 transmission to antibody neutralization *in vitro*. This assay will contribute to understanding the fundamental mechanisms of HIV-1 cell-to-cell transmission, allow evaluation of pre-existing or acquired HIV-1 resistance in clinical trials, and can be adapted to study other retroviruses.

**IMPORTANCE:**

HIV-1 can efficiently transmit from one cell to another but accurate quantification of this mode of transmission is still challenging. Here, we developed an ultrasensitive assay to measure HIV-1 transmission between cells and to evaluate HIV-1 escape from broadly neutralizing antibodies in primary human T cells. This assay will contribute to understanding the fundamental mechanisms of HIV-1 cell-to-cell transmission, allow evaluation of pre-existing or acquired HIV-1 resistance in clinical trials, and can be adapted to study the biology of other retroviruses.

## INTRODUCTION

HIV-1 replicates in host cells to produce new progeny that will then infect new target cells by one of two alternative modes. Typically, free viruses bud from infected cells and diffuse into biological fluids, where they are diluted, and circulate in the host until they encounter new permissive cells. In addition, an infected cell can directly contact a permissive cell to form virological synapses (VSs). Within VSs, budding virions are concentrated in close proximity to the membrane of an uninfected cell and can efficiently interact with the CD4 and CCR5/CXCR4 receptors to enter the new target cell (1). Mechanisms of HIV-1 cell-cell transmission usually involve changes in producer cells. These include the engagement of adhesion molecules on infected cells with related molecules on uninfected target cells and cytoskeleton reorientation toward cell-cell contact. However, despite the wealth of information, different aspects of HIV-1 cell-cell transmission, such as signaling and activation of target cells, membrane sites of the virion budding and fusion at VSs, and correlation between morphological events and HIV-1 infection, are still controversial or poorly understood (2).

**Fig 1:**
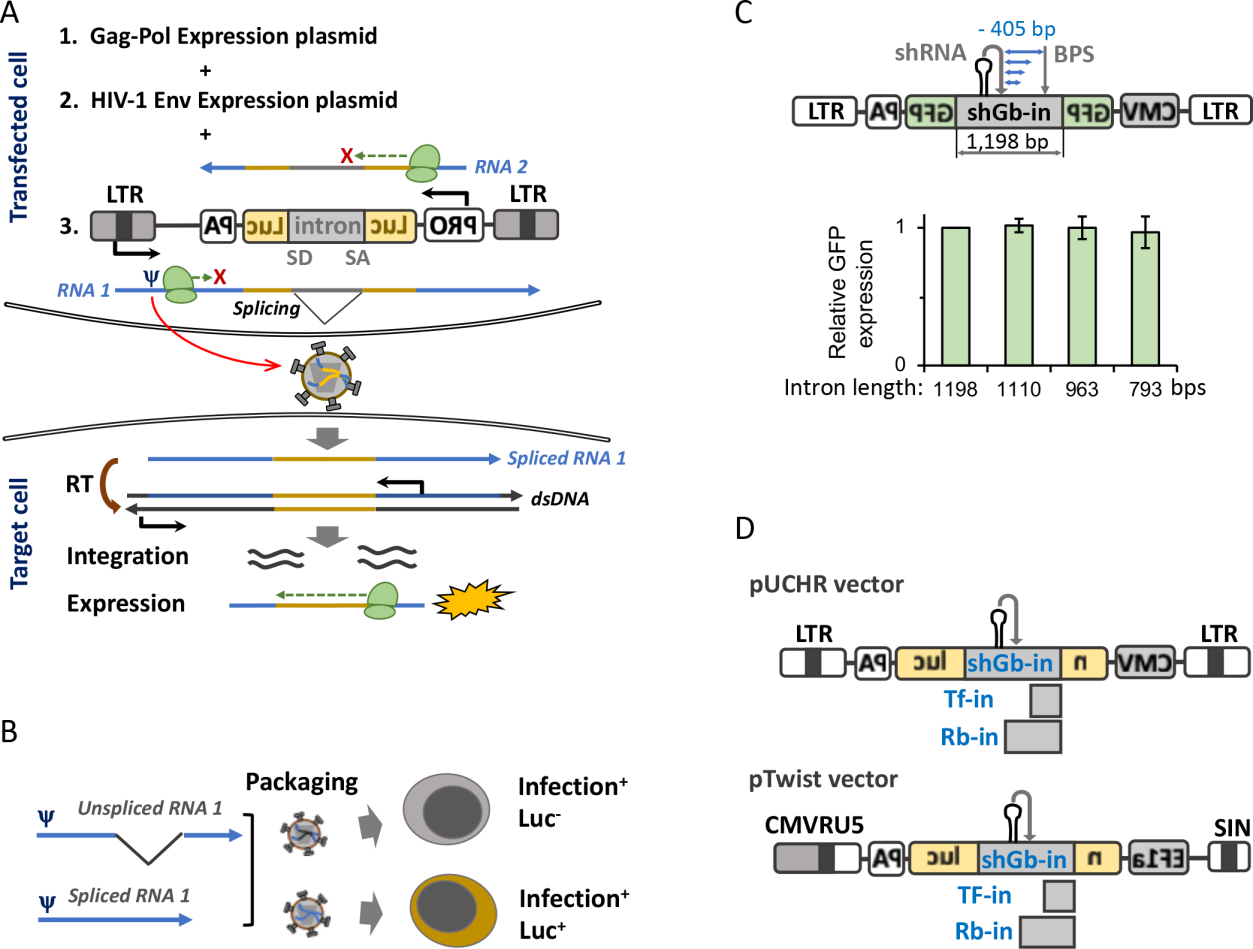
Design of cell-cell transmission reporter vectors. (**A**) A scheme of engineered elements in the reporter vector. These include the following blocks in the reverse orientation: promoter (PRO), 5′- and 3′-portions of reporter *luc* gene separated by an intron, and polyA (PA) signal (all shown by inverted letters). The splice-donor (SD) and the splice-acceptor (SA) sites flanking the intron are not reversed and therefore mRNA transcribed from the promoter cannot be spliced. Transfection leads to the transcription of two mRNAs: RNA 1 can be spliced but will not express the reporter protein because the reading frame is reversed; RNA 2 cannot be spliced and therefore will not express the reporter protein. Only RNA 1 can be packaged into virions due to the ψ signal and during productive infection, reverse transcriptase (RT) will synthesize (−) strand DNA containing the *luc* gene in the correct orientation, (+) strand DNA and integrates the double-stranded DNA into host chromosome where the Luc protein can be expressed from the promoter PRO. (**B**) One potential limitation of using the reporter vector is the packaging of unspliced RNA 1 into the nascent virions. This activity decreases the detection of cell-to-cell transmission since the resulting integrated provirus will still contain the intron and will not support Luc expression. (**C**) Top: a scheme of shortening of shRNA-modified human γ-globin intron (shGb-in) between shRNA target site and branch point site (BPS). Bottom: relationship between shGb-in length and reporter protein (GFP) expression. 293T cells were co-transfected with HIV-1 packaging vector, VSV-G expression plasmid, and GFPturbo-based shGb-in reporter vectors. Percentage of GFP+ cells was measured 2 days post-transfection by flow cytometry and normalized to GFP expression mediated by the original pUCHR-inGFPt reporter vector (in which intron length is 1,198 bp). (**D**) A scheme of new nanoluciferase (*nluc*)-based lentiviral vectors with three types of introns generated and tested in this study. Luc, luciferase; LTR, long terminal repeat; EF1a, elongation factor 1a-HTLV hybrid promoter; SIN, self-inactivating 3-LTR; shGb-in, shRNA-modified human γ-globin intron; TF-in, mouse TNFβ intron; Rb-in, ribozyme intron. Results shown in panel C are the mean ± SD of three independent experiments.

Accurate *in vitro* measurement of HIV-1 cell-cell transmission is challenging because of the difficulties to distinguish between infection of free virions and transmission of viruses between cells. To address these obstacles, several experimental approaches have been previously described. These include time course analysis of transmission modes (3, 4), shaking lymphocyte cocultures (5), and selection of experimental conditions that significantly favor cell-to-cell transmission in the absence of diethylaminoethyl dextran (6, 7). A second challenge for measuring cell-cell transmission is the separation of virus-producing cells from target cells under coculture conditions. Immunofluorescence staining of HIV-1 Gag transfer to target cells using microscopy or flow cytometry is often used to quantify the cell-cell transmission of HIV-1 in primary cells (5, 8, 9). In these methods, target cells are typically labeled with a fluorescent dye, and HIV-1 transmission between cells is detected by measuring HIV-1 Gag transfer to target cells. However, immunofluorescence methods do not necessarily reflect productive infections (10). An alternative method using fluorescent molecular clones such as iGFP (11) to monitor cell-cell transmission requires additional labeling of target cells. Conventional luciferase-based single-round or replication-competent HIV-1 assays express high levels of reporter protein in transfected cells, which are used to produce the viruses for transmission. This phenotype obscures the detection of newly infected target cells in coculture assays typically used for cell-cell transmission experiments *in vitro*. To overcome some of these limitations, we have previously developed the replication-dependent reporter vectors that confine the expression of the reporter protein to post-transmission stage and after reverse transcription of HIV-1 genome in the newly infected cell (Fig. 1A). These vectors commonly contain an expression cassette consisting of a promoter, an intron-containing reporter gene, and polyA signal. The complete cassette is inserted in the antisense orientation relative to the viral sequence (12). This specific design prevents the expression of active reporter protein in transfected (virus-producing) cells from the two mRNAs that are transcribed (Fig. 1A). Reporter expression from LTR-mediated mRNA transcript is blocked because the reporter gene is positioned in a reverse orientation, and reporter expression from the internal promoter (cytomegalovirus promoter[CMV])-mediated mRNA is blocked because the mRNA contains the complement splicing sites and therefore the intron cannot be efficiently spliced out. Only when the viral genome, which is transcribed from the LTR, is efficiently spliced, packaged into the nascent virion, and reverse transcribed during transmission to the target cell, the reporter protein is expressed (Fig. 1A). One drawback of this system is the dependency on splicing of the reporter gene in the viral genome since both spliced and unspliced mRNA can be packaged into the nascent virions, and the latter does not support reporter protein expression (Fig. 1B). Subsequent efforts optimized the intron in the reporter gene and engineered an internal anti-intron shRNA within the intron sequence to reduce unspliced mRNA packaging, creating in-GFPturbo and in-mCherry vectors that resulted in up to 80% spliced mRNA packaging in HIV-1 virions (13). However, the splicing efficiency for mRNA from similar vectors containing the *firefly luciferase* gene remained low and did not allow accurate and sensitive measurements of HIV-1 cell-cell transmission in human primary CD4^+^ T cells.

Importantly, HIV-1 cell-cell transmission exhibits varied degrees of resistance to broadly neutralizing antibodies (bnAbs) against HIV-1. These bnAbs target highly conserved regions of the envelope glycoproteins (Envs) and block viral entry of different HIV-1 strains (14–19). bnAbs are developed in a small fraction of people living with HIV-1 after several years of infection and some bnAbs prefer to neutralize specific Env conformations (20–22). Immunotherapy and antibody-mediated protection trials are currently evaluating bnAbs as treatment or prevention modalities, but the documented ability of HIV-1 cell-cell transmission to evade bnAbs *in vitro* is a significant concern for the application of bnAbs to medical interventions. Thus, a robust and reproducible assay to monitor cell-cell transmission in primary CD4^+^ T cells could provide new tools to understand the mechanisms of HIV-1 transmission between cells and to investigate mechanisms of HIV-1 resistance to bnAbs by cell-to-cell transmission.

Here, we designed and built a reversed intron reporter vector (designated in-Nluc) to measure HIV-1 cell-cell transmission, which is based on the *nanoluciferase* gene and depends on HIV-1 for replication. The splicing efficiency of mRNA transcribed from in-Nluc is higher than 99% and sensitivity is >10^4^-fold higher than the original firefly luciferase vectors. in-Nluc reporter quantitatively measures HIV-1 cell-to-cell transmission in different cell-coculture systems (e.g., from adherent 293T to Cf2Th-CD4/CCR5 cells, and between T cell lines), including peripheral blood CD4^+^ T cells. High dynamic range and low background enable accurate measurements of the inhibition of HIV-1 cell-cell transmission by neutralizing antibodies. in-Nluc will be a helpful tool both for the evaluation of HIV-1 resistance in clinical trials and for fundamental studies of the mechanisms of HIV-1 cell-to-cell transmission.

## RESULTS

### Design and development of cell-cell transmission vectors expressing nanoluciferase

Current lentiviral-based reporter vectors that contain reverse intron to detect cell-cell transmission include HIV-1 Rev response element for viral RNA nuclear export and a packaging signal (Ψ) for packaging the reporter RNA into virions. These requirements result in the packaging of RNA that contains either spliced or unspliced reporter gene into virions, decreasing the sensitivity of the assay that requires spliced RNA to express intact reporter protein in target cells (Fig. 1B). One approach to significantly increase packaging of RNA with spliced reporter gene into virions is to engineer anti-intron Mir30-shRNA *cis*-element within the reporter gene intron to facilitate the degradation of RNA-containing unspliced reporter gene (13). This approach is expected to degrade the cytoplasm RNA-containing unspliced reporter gene that escaped the splicing machinery in the cell nucleus and was exported to the cytoplasm. However, applying this strategy to reporter vectors containing the *firefly luciferase* gene (in-Luc-mR) has resulted, so far, in low splicing efficiency.

We designed and built an improved lentiviral-based reporter vector to efficiently and accurately measure HIV-1 cell-to-cell transmission by introducing the following modifications to current vectors (Fig. 1): (i) we replaced the firefly luciferase gene (*fluc*) with the nanoluciferase gene (*nluc*), increasing signal readout by 1–2 orders of magnitude (23, 24); (ii) we optimized the intron by testing three different introns within the *nluc* gene: (a) short-length version (793 bp) of shRNA-modified human γ-globin intron (shGb-in). We used the short version based on similar cell-cell transmission efficiencies of inGFP reporter vector with different shGb-in lengths (793–1,198 bp; Fig. 1C) (13); (b) intron II from the mouse TNFβ (Tf-in), which efficiently spliced out introns from the neomycin-resistance gene (25); and (c) the ribozyme self-splicing intron (group I intron) of *Tetrahymena thermophila* (Rb-in) (26); (iii) we selected the best potential sites for intron insertion to increase intron/exon boundaries that are accessible to the spliceosome complex and devoid of interfering secondary structures as previously described (13). Specifically, we inserted the shGb and Tf introns downstream to CAG sequences of the reversed *nluc* gene and optimized predicted RNA folding by mfold (13). Variants containing intron(s) insertion with the highest numbers of unpaired nucleotides at splice donor (SD), splice acceptor (SA) sites, and the adjacent Nluc sequence are highlighted in Fig. S1; and (iv) we introduced several nonsense mutations at the 3′-coding region of the reversed *nluc* gene (shown in purple in Fig. S1) to reduce the pairing with SD, as well as other nonsense mutations to generate complementary sequence to the internal guide sequence of Rb (all introduced mutations are highlighted in Fig. S1), which are required for efficient ribozyme splicing (27). We applied this design to build two types of plasmids: (i) pUCHR-based second-generation lentiviral vector containing the CMV promoter to mediate nanoluciferase expression, and (ii) pTwist-based third-generation lentiviral vector containing the EF1a-HTLV hybrid promoter to mediate nanoluciferase expression (Fig. 1D).

### Transduction and splicing efficiencies of new reporter vectors in 293T cells

**Fig 2:**
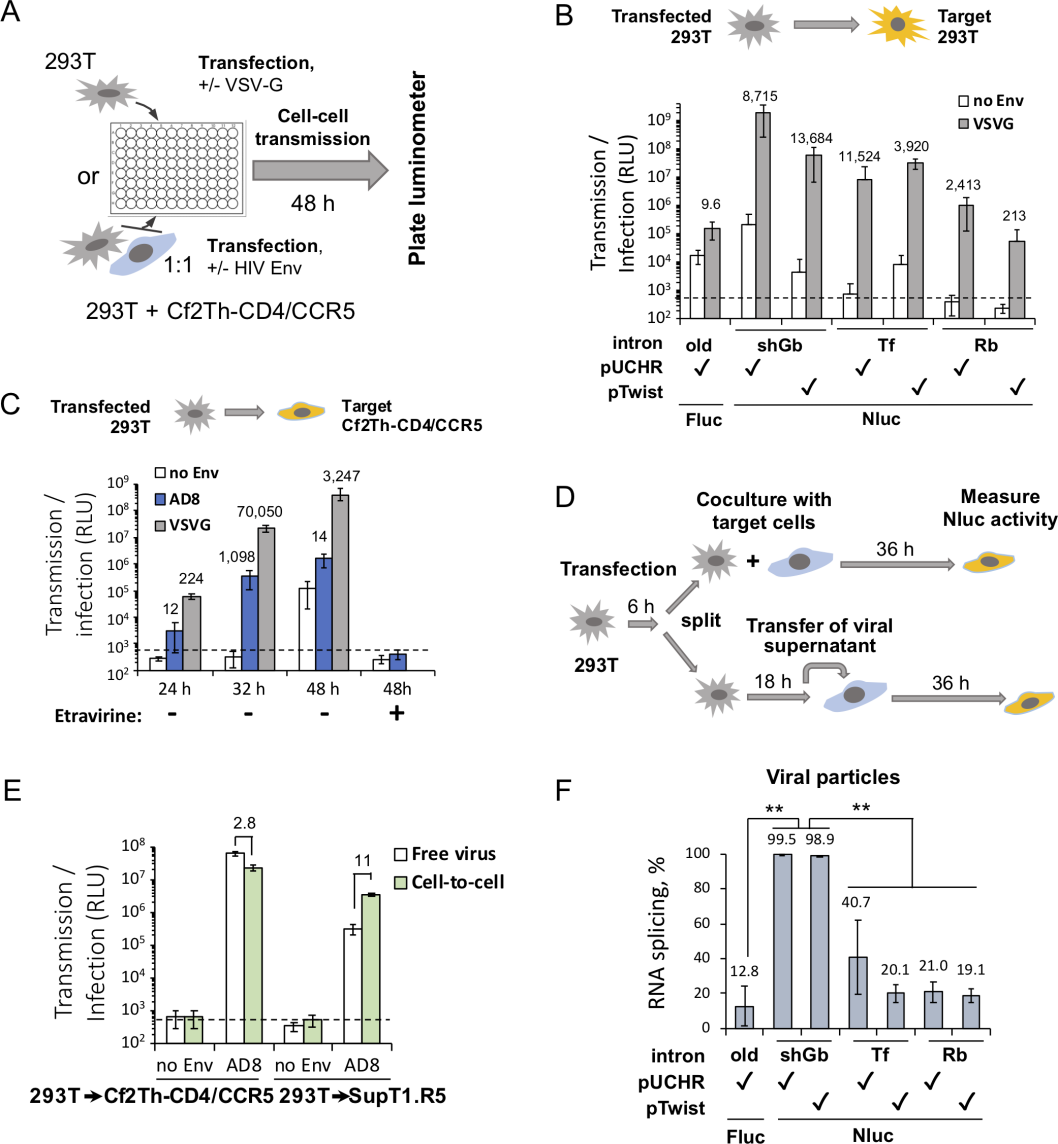
Optimizing the in-Nluc reporter vectors for the detection of cell-cell transmission in 293T cells. (**A**) A scheme of assay setup for the detection of cell-cell transmission in 293T cells or 293T-Cf2Th-CD4/CCR5 coculture. (**B**) Levels of HIV-1 cell-cell transmission to 293T cells, measured using different vectors and *luc* genes, 48 h after co-transfecting 293T cells with psPAX2 packaging and indicated reporter plasmids with or without VSV-G expression plasmid. (**C**) Levels of cell-cell transmission from 293T to Cf2Th-CD4/CCR5 cells measured by pUCHR-shGb-inNluc vector at indicated time points post-transfection in the presence or absence of indicated Envs and the non-nucleoside reverse transcriptase inhibitor Etravirine. (**D**) A scheme of experimental setup for the comparison of free HIV-1 infection and viral cell-to-cell transmission for viruses produced by 293T cells. (**E**) Levels of free HIV-1 infection and viral cell-to-cell transmission in the presence or absence of HIV-1_AD8_ Env. (**F**) Ratio of spliced and unspliced reporter RNAs packaged into virions was quantified by RT-quantitative PCR (see Materials and Methods for details). Dashed line (panels C and E) indicates background level of Nluc activity. Numbers indicate fold change (panels B,C and E) or the percentage of spliced RNA (panel F). RLU, relative light units. Results (panels B, C, E, and F) are representative of one out of three independent experiments. Student’s *t* test **P value < 0.01.

We tested the ability of our vectors to detect cell-cell transmission in 293T cells and in the coculture of 293T and Cf2Th-CD4/CCR5 cells. Viral transmission was initially evaluated in the presence, and as a control, absence of the vesicular stomatitis virus G protein, which mediates efficient entry into a variety of cells (Fig. 2A). pUCHR-shGb-inNluc resulted in high cell-cell transmission readout but elevated background, whereas pUCHR-Tf-inNluc exhibited the highest signal/noise ratio. Notably, the newly designed pUCHR-shGb-inNluc was ~4 orders of magnitude more sensitive than the original pUCHR-inLuc-mR vector but displayed increased Env-independent activity (Fig. 2B). Similar results were obtained using HIV-1_AD8_ Envs to mediate cell-cell transmission in a coculture of 293T (producing) cells and Cf2Th-CD4/CCR5 (target) cells (Fig. 2C). Low transfection efficiency into Cf2Th-CD4/CCR5 most likely led to dominating cell-cell transmission from 293T to Cf2Th-CD4/CCR5 cells. As expected, HIV-1_AD8_ Envs were less efficient than VSV-G in mediating viral entry and resulted in a decreased signal/noise ratio. Unexpectedly, background levels could be significantly decreased by the non-nucleoside reverse transcriptase inhibitor Etravirine. This pattern of inhibition suggested that the source of background was related to Env-independent cell-cell transmission into target cells rather than background Nluc expression in the producing cells in this coculture. The mechanism of this Env-independent transmission is currently unknown. However, several factors could potentially contribute to the detection of Env-independent infection events in these cells, including prolonged incubation, traces of transfection reagent, high efficiency of 293T transfection, which is expected to generate a high number of viral particles without Envs, and ultra-sensitive detection using the nanoluciferase reporter protein. Interestingly, a similar observation has been previously reported in epithelial cells (28). Importantly, we did not detect Env(-) infection/transmission in lymphoid cell lines or primary cells (next sections), which are more relevant to authentic, *in vivo* replication of HIV-1. We compared the contribution of infection by free HIV-1 virions and the contribution of viral cell-to-cell transmission, both produced by 293T cells, to the overall readout as previously described (3). HIV-1 cell-cell transmission was ~11-fold more efficient than free HIV-1 infection when transmission was measured from 293T cells to lymphoid cell line SupT1.CCR5, but cell-cell transmission was approximately threefold less efficient than free HIV-1 infection when we used the adherent target cells Cf2Th-CD4/CCR5 (Fig. 2E). Thus, we focused our subsequent work (next sections) on HIV-1 cell-cell transmission in human T cells, which more closely reflects the ability of HIV-1 to replicate *in vivo*. We determined the efficiency of reporter RNA splicing and packaging by measuring the ratio of spliced to total RNA packaged into virions. Splicing efficiencies generally correlated with the cell-cell transmission measurements (Fig. 2F).

Overall, shGb intron-based vectors supported high-efficiency splicing of Nluc RNA, high sensitivity in a 96-well format (2 × 10^4^ of 293T transfected with 22 ng of plasmid DNA), and low background in producing cells.

### Quantification of HIV-1 cell-cell transmission and free virus infection in human T cell lines with the new reporter vectors

**Fig 3:**
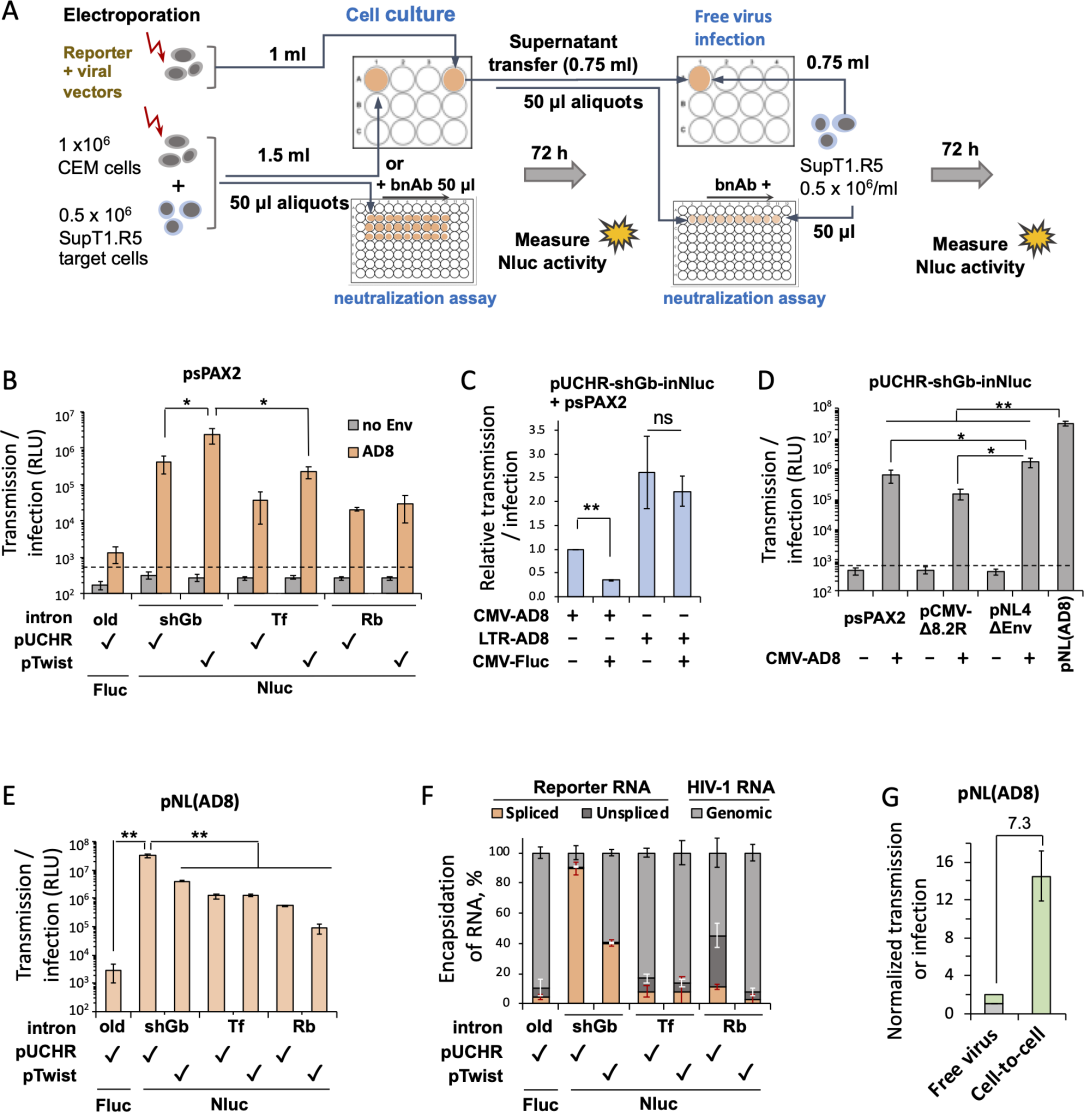
Cell-cell transmission in CD4^+^ T cell lines detected by the inNluc reporter vectors. (**A**) A workflow for the quantification of cell-cell transmission from CEM to SupT1.CCR5 cells by the inNluc reporter vectors and, in parallel, of free virus infection under identical conditions. (**B**) Cell-cell transmission measured by different reporter vectors co-transfected with the packaging (psPAX2) and HIV-1_AD8_ Env-expressing plasmids, in which transgenes are transcribed from the CMV promoter. (**C**) Effects of CMV-driven transgene expression on the cell-cell transmission readout. Cells were co-transfected with pUCHR-CMV-shGb-inNluc reporter vector, packaging plasmid psPAXS2 and different HIV-1_AD8_ Env-expressing plasmids with or without Fluc-expressing plasmid pGL3-CMV. 3′ end of the *env* gene in LTR-AD8 is driven from NL4-3 *env*. (**D**) Effects of different packaging vectors or full-length HIV-1 with deleted *env* on the cell-cell transmission readout. (**E**) Measurements of cell-cell transmission mediated by the molecular clone pNL4(AD8) using the indicated reporter vectors. (**F**) Ratio of spliced reporter RNA, unspliced reporter RNA, and viral genomic RNA packaged into virions produced in 293T cells after co-transfection with pNL(AD8) and the indicated reporters. (**G**) In parallel estimate of HIV-1_AD8_ cell-cell transmission and free virus infectivity. After a 3-day incubation of the cell-cell transmission assay, cells were centrifuged. Half of the supernatant containing free viruses was used to infect fresh SupT1.R5 target cells, and free virus infection was measured after an additional 3 days. Readout for half of the supernatant was set to 1 (gray bar), and the readout adjusted for the total volume is shown as a green bar (both for free virus infection). Cell-cell transmission was measured using the centrifuged cells on day 3 and the readout was normalized to virus-free infection. Fold change between the cell-cell transmission and free virus infection (after adjustment for total volume) is indicated. ns, not significant; *P value < 0.05; and **P value < 0.01; both *P* values were calculated by two-tailed Student’s *t*-test.

We next tested the ability of our reporter system to measure cell-cell transmission in T-cells, which more closely reflects the authentic replication of HIV-1 in primary CD4^+^ T cells. We used CEM as producing cells and SupT1.CCR5 as target cells (in a 12-well and 96-well format) to test different combinations of reporter, packaging, and Env-expression plasmids (Fig. 3A). Vectors containing the shGb intron co-transfected with Gag-Pol-expressing plasmid (psPAX2) and HIV-1 Envs exhibited the highest level of Nluc expression. However, in contrast to 293T cells, the pTwist-based plasmid was more sensitive than the pUCHR-based plasmid (Fig. 3B). We detected only minimal background in the T-cell coculture system. Interestingly, the co-transfection of the pUCHR-shGb-inNluc reporter with a full-length HIV-1 vector pNL4ΔEnv containing only a frame-shift mutation in *env* gene led to a higher level of cell-to-cell transmission in T cells compared to two versions of packaging plasmids (pCMV-dR8.2 and psPAX2). Even higher readout was measured when we used a plasmid containing a full-length molecular clone pNL4(AD8) (Fig. 3D). Thus, despite potential competition of the reporter RNA with the viral genomic RNA (from the full-length HIV-1 plasmids), the reporter RNA was still efficiently packaged into virions. Cell-cell transmission detected by pUCHR-shGb-inNluc exhibited a higher readout among the different inNluc reporter vectors when co-transfected with pNL(AD8) (Fig. 3E). The overall readout correlated with the efficiency of the spliced reporter RNA to be packaged into HIV-1 virions (Fig. 3F, orange histograms). Of note, high readout when using the replication-competent pNL(AD8) may potentially be the result of a second-round infection. Nevertheless, the second round of infection would require (i) first round of co-infection with two viruses: replication-competent NL(AD8) and a virus carrying the inNluc reporter, or (ii) recombination between two different genomic viral RNA [one of NL(AD8) and a second of the inNluc reporter]. While shGb intron splicing was equally efficient when incorporated in pUCHR and pTwist vectors (Fig. 2F), the pUCHR provided about twofold higher level of total RNA incorporation into virions compared to analogous pTwist vector. More efficient incorporation led to higher readout. Consistent with previous reports (5, 8), free virus infection of HIV-1_AD8_ was ~7.3-fold less efficient (Fig. 3G) than cell-cell transmission.

### Detection of HIV-1 cell-cell transmission between primary human CD4^+^ T cells

**Fig 4:**
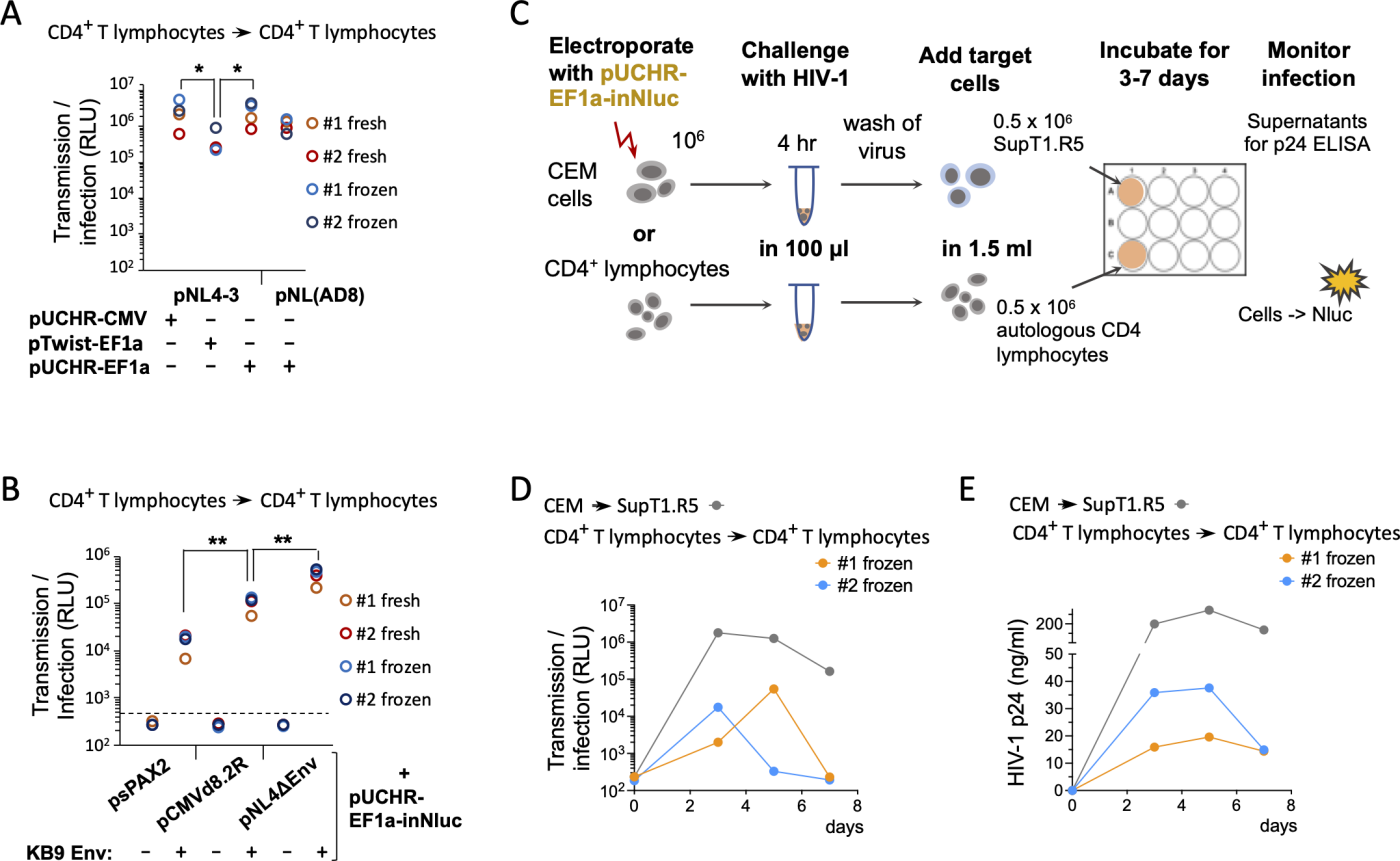
Detection of HIV-1 cell-cell transmission in human primary CD4^+^ T cells by different reporter vectors. (**A and B**) Cell-cell transmission in primary CD4^+^ T cells after co-transfection of different reporter vectors containing the shGB-inNluc gene together with plasmids containing full-length HIV-1 provirus (**A**) or with plasmids containing HIV-1 packaging genes (**B**). Data in panels **A** and **B** were obtained using fresh or frozen/thawed PBMCs from two donors (#1 and #2). (**C**) A scheme of experimental setup for monitoring cell-cell transmission during HIV-1 replication. (D and E) Kinetics of cell-cell transmission as detected by Nluc expression during HIV-1 replication (**D**) and kinetics of HIV-1 replication in the same cells detected by p24 release into the supernatant (**E**). CEM or primary CD4^+^ T cells were transfected with pUCHR-EF1a-shGb-inNluc reporter vector and then infected with 300 ng of HIV-1_NL4-3_. *P value < 0.05 and **P value < 0.01; both *P* values were calculated by two-tailed Student’s *t*-test.

We next selected the two most efficient reporter vectors (containing the shGb intron in pUCHR and pTwist backbones) and tested their ability to measure cell-cell transmission in primary CD4^+^ T cells. For these experiments, we generated an additional pUCHR-based vector, designated pUCHR-EF1a-inNluc, in which we replaced the CMV promoter with EF1a-HTLV-1 hybrid promotor to study the effect of promoter on the measurements in primary cells. We isolated CD4^+^ T cells from two donors, and activated and then electroporated the cells as previously described (29). All subsequent steps were performed as described for the T cell lines, but transfected cells were cocultured with autologous primary CD4^+^ T cells. Both pUCHR-shGb-inNluc and pUCHR-EF1a-inNluc vectors, containing either CMV or EF1a-HTLV-1 promoter, co-transfected with pNL4-3 (a plasmid that contains the NL4-3 molecular clone; X4 tropic), resulted in a high and comparable readout of cell-cell transmission (Fig. 4A). In comparison, pTwist-based vector demonstrated ~6.7-fold lower level of Nluc activity in primary CD4^+^ T cells. Lower activity was consistent with the low efficient packaging of RNA transcribed from the pTwist reporter into HIV-1 virions (Fig. 3F). We also detected comparable cell-cell transmission of NL4-3 and an R5-tropic molecular clone NL(AD8) despite typically lower levels of CCR5 than CXCR4 expected on activated CD4^+^ T cells. In addition, we detected cell-cell transmission using single-round HIV-1 vectors in primary CD4^+^ T cells when the reporter plasmid was co-transfected with a packaging plasmid and dual-tropic HIV-1_KB9_ Envs. Overall, our system exhibited minimal background without HIV-1 Envs for all packaging plasmids (Fig. 4B), and we detected a differential readout of cell-cell transmission using different packaging plasmids that ranked as psPAX2 < pCMV-d8.2R < pNL4-3. Unlike the results with T cell lines, the use of pCMV-d8.2R packaging vector in primary cells led to detection of a higher level of HIV-1 cell-cell transmission compared with the use of psPAX2 vector . This may reflect the requirement of accessory genes for efficient HIV replication in primary cells, which are deleted in the psPAX2 vector.

To study HIV-1 cell-cell transmission after virus inoculation, we next investigated whether RNA from the reporter vector can be efficiently incorporated into viral particles during HIV-1 replication in cells containing the cell-cell transmission reporter vector (and not only when the reporter and viral vector are co-transfected by electroporation into producing cells). CEM cells or primary CD4^+^ T cells were transfected with the reporter vector pUCHR-EF1a-inNluc only by electroporation. Transfected cells were then incubated with 300 ng of concentrated HIV-1_NL4-3_, washed, and mixed with the related target cells (SupT1.CCR5 or primary CD4^+^ T cells; Fig. 4C). Our reporter vector detected cell-cell transmission in cell lines and primary CD4^+^ T cells within 3–5 days after inoculation. Nevertheless, the detection was 1–2 orders of magnitude lower than the co-transfection of all viral vectors. Nluc expression in target cells correlated with p24 in the supernatants of infected cells (Fig. 4E).

In conclusion, the high sensitivity of the new in-Nluc vectors provides robust detection of HIV-1 cell-cell transmission in activated primary CD4^+^ T cell cocultures. We detected cell-cell transmission after co-transfecting with either HIV-1 molecular clones or single-round replication vectors, or after a combination of transfecting cells and then inoculating transfected cells with HIV-1 virions.

### Sensitivity of cell-cell transmission to broadly neutralizing antibodies against HIV-1 Envs

**Fig 5:**
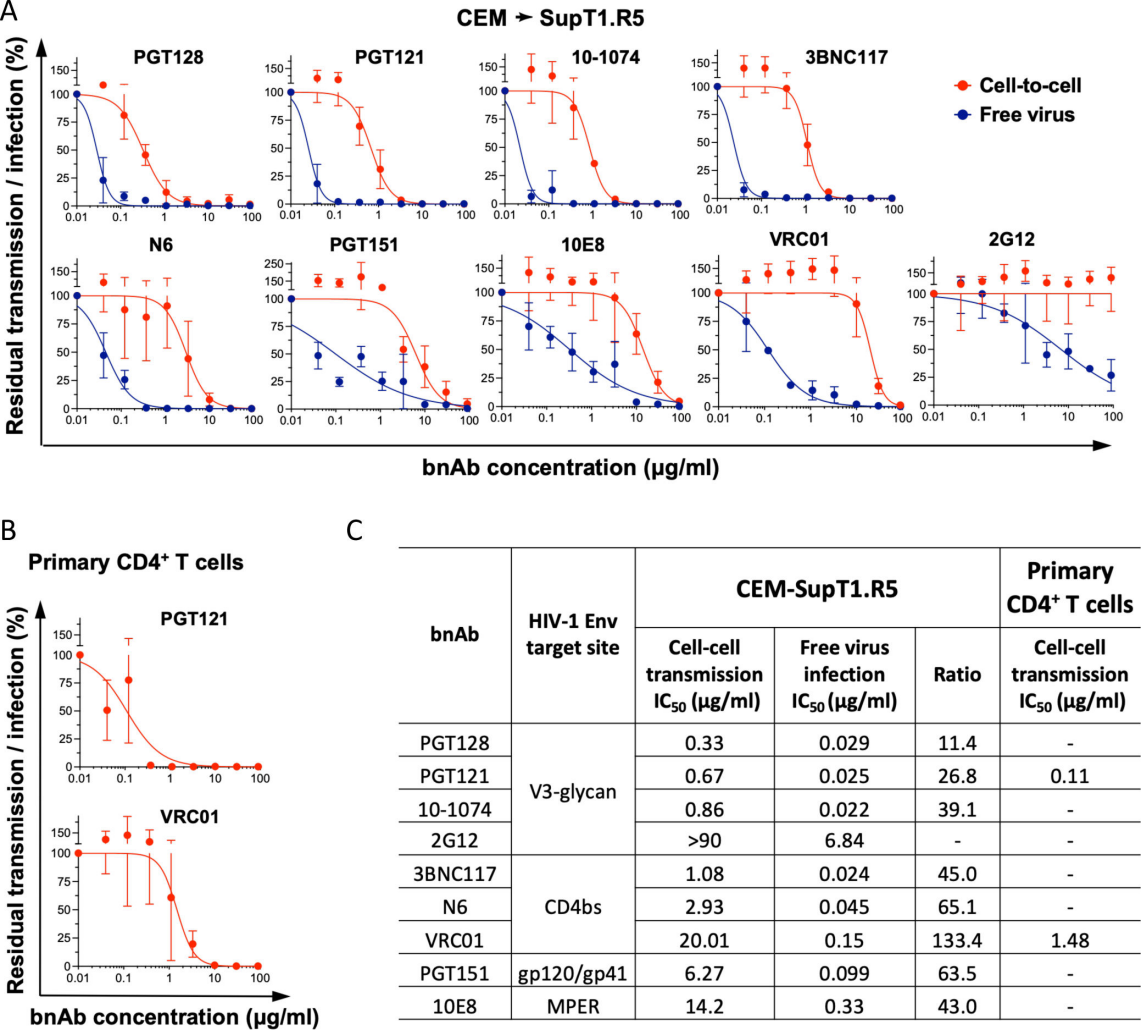
Sensitivity of HIV-1 cell-cell transmission in CD4^+^ T cells to bnAbs. (**A**) Dose-response curves of HIV-1 cell-to-cell transmission and free virus infection in CEM-SupT1 cell system in the presence of nine individual bnAbs targeting different sites of HIV-1_AD8_ Envs. CEM cells were co-transfected with pUCHR-EF1a-shGb-inNluc, pNL4ΔEnv, and LTR-AD8 plasmids and used to measure sensitivity of cell-cell transmission and free virus infection to antibody neutralization as described in the Materials and Methods section. Assays on T cell lines were performed twice in duplicates, and data are presented as average values with the standard deviations. (**B**) Sensitivity of cell-to-cell transmission between primary CD4^+^ T cells to the indicated bnAbs. Activated CD4^+^ lymphocytes were electroporated with the same reporter vector and pNL(AD8) plasmid. A mixture of transfected and non-transfected autologous CD4^+^ T cells was added to the diluted bnAbs in one replica. Sensitivity of HIV-1 cell-to-cell transmission to each bnAb was measured using CD4+ lymphocytes from donor #1 and #2 and presented as the average values with standard deviations. (**C**) Calculated IC_50_ values for HIV- 1_AD8_ sensitivity to each bnAbs tested with free virus or during cell-to-cell transmission. Results are the average ± SD calculated from at least two independent experiments, each performed in one (primary CD4^+^ T cells) or four (T cell lines) replicates.

We detected cell-cell transmission in T cells with high sensitivity and low background levels using the pUCHR-EF1a-inNluc reporter vector. These properties provided a wide dynamic range for accurate quantification of cell-cell transmission sensitivity to neutralization by bnAbs. We therefore next measured HIV-1 transmission sensitivity to bnAbs that target the following HIV-1 Env vulnerability sites: CD4 binding site, gp120 V3 glycan, gp120-gp41 interface, and gp41 membrane proximal external region. We compared the inhibition efficiency of HIV-1 transmission (expressed as IC_50_; half-maximal inhibitory concentration) to the inhibition efficiency of free viruses present in the supernatant of these cells. To produce free viruses under identical conditions, we transfected CEM cells without target cells and incubated them in parallel to the CEM-SupT1.CCR5 coculture for a similar 3-day period. We then added virion-containing supernatant to diluted bnAbs and SupT1.CCR5 target cells (a single replicate) and incubated the plate for 3 days. Consistent with several previous reports, bnAbs were significantly less efficient in blocking cell-cell transmission than neutralization of free virions in supernatant (6–8, 30, 31). Overall, we measured at least 1 order of magnitude reduced neutralization efficiency of cell-cell transmission for all bnAbs tested (Fig. 5A and C). In particular, cell-cell transmission was most resistant to VRC01 (IC_50_ = 20 µg/mL) while free viruses were still sensitive to VRC01 with an IC_50_ = 0.12 µg/mL. Thus, VRC01 was >2 orders of magnitude more efficient in neutralizing free viruses than blocking transmission between cells. We also quantified the sensitivity of HIV-1 cell-to-cell transmission in primary CD4^+^ T cells to bnAbs (Fig. 5B). As expected, we detected lower signal in primary cells than in T cell lines, but the general pattern was similar with relative resistance of cell-cell transmission to bnAb inhibition (Fig. 5C and Table S3).

Thus, our new reporter vector can robustly measure the sensitivity of HIV-1 cell-to-cell transmission to bnAbs in both CD4^+^ T cell lines and primary CD4^+^ T cells.

## DISCUSSION

Quantitative measurement of HIV-1 cell-to-cell transmission using reporter vectors is challenging due to multiple obstacles. The use of reversed and intron-inactivated reporter genes (e.g., *fluc*) allows distinguishing between transfected and newly infected cells in cell cocultures. However, currently available reporter vectors that use the reverse firefly luciferase gene and contain an intron have substantial drawbacks due to the packaging of the HIV-1 genome containing the unspliced reporter gene (12, 13). These vectors allow measurements of HIV-1 cell-cell transmission between lymphoid cell lines, as well as in cocultures of Raji cells and PBMCs. However, their use for the detection of transmission between primary CD4^+^ T cells is still limited (13). Alternative in-*Gluc* reporter vectors are based on secreted *Gaussia* luciferase. They were initially designed for the detection of cell-cell transmission of murine leukemia virus (MLV) (32) and subsequently adapted for HIV-1 research (3). These vectors exhibit 3 orders of magnitude higher signal compared to the firefly luciferase (33), but high signal is associated with elevated background levels due to the oxidation of the coelenterazine substrate by components in fetal bovine serum (FBS) typically present in culture media (34). As a result, the signal-to-background ratio using these vectors is reduced and they have been mainly used to measure cell-cell transmission in highly transfectable 293T cells to highly permissive cell lines 293T.CD4.CXCR4, HeLa TZM-bl, MT4 (3), or A3.01.CCR5 (7).

Here, we addressed current limitations on cell-cell transmission reporter vectors by designing and building an ultra-sensitive assay to measure HIV-1 cell-to-cell transmission. We optimized the length of shRNA-containing intron without compromising splicing efficiency. We also codon optimized sequences that are predicted to be involved in RNA secondary structure at SA and SD sites to prevent potential interference. This approach led to very efficient *nluc* gene splicing (Fig. 2F) and, together with superior brightness and minimal background, provided unprecedent high sensitivity and wide dynamic range of the NLuc reporter vectors. As a result, in CEM-SupT1 coculture, co-transfection of pUCHR-shGb-inNluc with pNL4(AD8) generated a signal/noise ratio of >80,000 (Fig. 3D). A combination of several factors could contribute to high sensitivity of detection of HIV-1 cell-cell transmission using the molecular clone pNL4(AD8) compared to transmission detected using Env-expressing plasmids. These include Env expression levels, accessory viral proteins expressed in producing cells, multiple rounds of HIV-1 replication during 3-day cell coculture, and more efficient encapsulation of spliced reporter RNA (Fig. 3F). High sensitivity and wide dynamic range allowed us to robustly measure the sensitivity of HIV-1 cell-cell transmission to neutralizing antibodies using CD4^+^ T cells in 96-well plate format. Moreover, we could compare this activity side-by-side with HIV-1 sensitivity (free virus) to neutralizing antibodies using the same cell system (Fig. 5).

Our improved and highly sensitive technology can immediately be applied to answer important research questions. As our reporter vector can detect HIV-1 replication in cocultures of primary CD4^+^ T cells (Fig. 4A and B), this system can be utilized to measure the ability of different HIV-1 strains from people living with HIV-1 (PLWH) to transmit between their primary CD4^+^ T cells *ex vivo*. In most treated PLWH, current anti-retroviral therapy decreases HIV-1 viral load to undetectable levels. Thus, one approach to potentially use our system would be to isolate CD4^+^ T cells from PLWH, transfect them with the NLuc reporter vector with subsequent activation of HIV-1 replication, and measure the cell-cell transmission efficiency of different clinically relevant HIV-1 Envs.

More importantly, our new ultrasensitive tool can accurately measure the sensitivity of cell-cell transmission to antibody neutralization. Due to broad coverage and high potency, different bnAbs have been or are currently being tested as therapeutic and prevention modalities in several completed and ongoing clinical trials. However, in some cases, despite high levels of bnAbs (i.e., VRC01 or VRC07) in the blood of treated patients, HIV-1 is still rebounding. Notably, the recrudescing strains are still sensitive *in vitro* to VRC01 or VRC07 and they do not exhibit any evidence of viral evolution to resist bnAbs (35, 36). One potential mechanism of HIV-1 resistance may be increased ability to transmit between cells *in vivo* (37). This process is relatively resistant *in vitro* to most bnAbs and particularly resistant to VRC01 neutralization (6–8, 38). Resistance may be related, at least partially, to limited penetration of antibodies to the VS (39). Thus, we are now in the process of obtaining resistant HIV-1 strains from immunotherapy clinical trials to study their ability to transmit between primary CD4^+^ T cells and to investigate the relationship between HIV-1 resistance to bnAbs and cell-cell transmission efficiency *ex vivo*.

In addition, the application of the reporter vectors is specific but not limited to HIV-1. They can be applied for the evaluation of humoral response against other viruses, specifically, SARS-CoV-2 whose cell-to-cell transmission has been reported to be resistant to neutralization (40, 41). Moreover, the reporter expression cassette (EF1a-shGb-inNluc-pA) can be subcloned into the other retroviral vectors, such as vectors supporting SIV (42), HTLV-I and MLV, to study cell-to-cell transmission at a new level of sensitivity. The algorithm for shGb intron insertion described here can be applied to create different selection genes with a high level of sensitivity to detect, for instance, retro-transposition events.

Overall, our improved reporter will provide new opportunities to study the mechanisms of viral cell-cell transmission, categorize different HIV-1 strains according to their cell-cell transmission, and investigate the cell-cell transmission sensitivity to antibody neutralization. These directions studied in primary cells are expected to provide new insights into relevant and complex biological processes.

## MATERIALS AND METHODS

### Cell cultures, primary cell isolation, and activation

Human embryonic kidney (HEK) 293T cells were obtained from the American Tissue Culture Collection, Cf2Th-CD4/CCR5 cells stably expressing human CD4 and CCR5 were kindly provided by Joseph Sodroski (Dana-Farber Cancer Institute), CEM CD4^+^ cells were obtained from the NIH AIDS Reagent Program, and SupT1.CCR5 (SupT1.R5) cells stably expressing the human CCR5 coreceptor were a kind gift from James Hoxie (University of Pennsylvania). Trima Cones containing concentrated human leukocytes from freshly collected blood were purchased from Innovative Blood Resources (Minneapolis, USA). Trima cone cells were diluted 1:3 with PBS, and the peripheral blood mononuclear cells (PBMCs) were isolated on the density gradient of lymphocyte separation medium 1077 (PromoCell, Heidelberg, Germany) using SepMate 50 mL tubes (Stemcell Technologies, USA) and then directly used or frozen in fetal bovine serum (Gibco) containing 5% dimethyl sulfoxide (DMSO; Sigma, USA). Before using in downstream applications, frozen PBMCs were thawed and incubated in a culture medium for 1 day. CD4^+^ T cells were isolated from fresh or frozen PBMCs by negative selection using the EasySep human CD4^+^ T cell isolation kit according to the manufacturer’s instructions (Stemcell Technologies, USA) and activated with CD2/CD3/CD28 magnetic beads (Miltenyi Biotec, Germany) for 48 h. Immediately prior to electroporation, the activating beads were removed from CD4^+^ lymphoblasts using the magnet. 293T and Cf2Th-CD4/CCR5 cells were cultured in Dulbecco’s modified Eagle’s medium (DMEM) (Sigma-Aldrich, USA) with 10% FBS, 2 mM glutamine, 100 U/mL penicillin, and 100 µg/mL streptomycin (all from Gibco, ThermoFisher). CEM and SupT1.CCR5 cells were maintained in RPMI 1640 medium containing 10% FBS, 2 mM glutamine, 100 U/mL penicillin, and 100 µg/mL streptomycin. Activated human CD4^+^ T cells were cultured in RPMI-1640 growth medium supplemented with 50 IU/mL of recombinant human interleukin-2 (IL-2) (Miltenyi Biotech, Germany). Cells were maintained at low passage and tested periodically for mycoplasma contamination.

### Plasmid construction

We optimized the length of the intron of the human γ-globin gene by generating three short PCR-amplified fragments using separately three forward primers, each combined with the common reverse primer, as indicated in Table S1, and pUCHR-inGFPt-mR plasmid (13) containing the full-length intron as a template. The resulting three PCR fragments of 104, 251, and 421 bp were separately cloned back into the pUCHR-inGFPt-mR plasmid via the XbaI/SpeI restriction sites. The nano luciferase-based HIV-1 reporter vectors pUCHR-inNluc and pTwist-inNluc containing shortened γ-globin intron with shRNA (shGb), intron from the mouse TNFβ gene (Tf), and *Tetrahymena* ribozyme intron (Rb) were designed as described in the Results section by Gene Universal (DE, USA). The sequences of expression cassettes for each reporter vector are provided in Fig. S1. To generate the pUCHR-EF1a-inNluc plasmid, we subcloned the hybrid EF1a-HTLV promoter from the pTwist-Tf-inNluc into pUCHR-inNluc plasmid, replacing the CMV promoter (see Fig. S2). The pTwist-Tf-inNluc plasmid was digested with Sal I, blunted, and then digested with Nhe I; the resulting digested insert was subcloned into the SmaI/NheI sites of pUCHR-inNluc. Plasmids containing the molecular clones HIV-1_NL4-3_ (pNL4-3), HIV-1_NL4-AD8_ [pNL(AD8)], and the packaging plasmid psPAX2 were obtained from the NIH AIDS Reagent Program; the packaging vector pCMV-dR8.2 was from Addgene. Plasmid containing HIV-1_NL4ΔEnv_ (pNL4ΔEnv) sequence, which carries a frame-shift mutation due to re-ligation of Nde I blunted ends in *env* gene, was generated by digesting NL4-3 Gag-iGFP ΔEnv plasmid (NIH AIDS Reagent Program) with Sal I/Xho I and cloning the resulting DNA fragment into the same restriction sites of pNL4-3 plasmid. The Env expression plasmids pcDNA 3.1-AD8-M, pSVIIIe7-AD8, pcDNA 3.1-KB9, and pCMV-VSVG (expression vector for protein G of vesicular stomatitis virus) were kindly provided by Joseph Sodroski (Dana-Farber Cancer Institute). The firefly luciferase expression plasmid pGL3-CMV was purchased from Promega (Madison, WI, USA). All plasmids used in the study are listed in Table S4.

### Quantification of spliced and unspliced reporter RNA packaging

To determine the splicing efficiency, 293T cells (5 × 10^5^ cells per well of a 6-well plate) were co-transfected with 0.35 µg of psPAX2 or pNL(AD8) and 0.45 µg of a reporter plasmid using Effectene transfection reagent (Qiagen, USA). The next day, the culture medium was removed, cells were gently washed with warm media once, and incubated in 2 mL of the medium for another day. Supernatants were collected, centrifuged at 3,000 × *g* for 5 min to remove cell debris, and further centrifuged (in 1.5 mL tubes) at 18,000 × *g* for 2.5 h to isolate viral particles. Viral RNA was isolated from the particles using the QIAamp DSP Viral RNA Mini Kit (Qiagen, USA) and treated with RNase-free DNase I (New England Biolabs) to remove traces of the plasmid DNA. All purified viral RNA was reverse transcribed using random hexamer and SuperScript III reverse transcriptase (Invitrogen, USA) for 1 h at 50°C after a short 10-min preincubation at 25°C. cDNA amount was assessed by quantitative PCR (qPCR) using GoTaq qPCR Master Mix (Promega, USA), 0.4 µM of each primer, and 1–5 ng of cDNA template. The primers used for the amplification of unspliced reporter RNAs (intron), spliced reporter RNAs, and HIV-1 genomic RNA (conserved Gag region) are shown in Table S2. PCR was performed using CFX-96 Real-Time PCR Detection System (Bio-Rad, USA) at the following settings: one cycle of denaturation at 95°C for 2 min and 40 cycles of amplification (95°C for 7 s, 58.4°C for 15 s, and 60°C for 15 s). Data were collected and analyzed by using CFX-96 software. The levels of spliced and unspliced reporter RNAs in viral particles were quantified for each transfer vector. The efficiency of RNA splicing was expressed as a percentage of the ratio of spliced to the total RNA (spliced and unspliced) and was calculated by the formula:


$$\frac{1}{1+2^{A-B}}\times 100,$$

where *A* is a threshold cycle (*C_T_*) for spliced RNA, and B is *C_T_* for unspliced RNA (13). When molecular clones were used, a similar calculation was performed to determine the percentage of spliced reporter RNA relative to the total (spliced, unspliced reporter, and viral genomic) RNAs packaged into HIV-1 virions


$$\frac{1}{1+2^{A-B}+2^{A-C}}\times 100,$$

percentage of unspliced reporter RNA relative to a total of three RNAs


$$\frac{1}{1+2^{B-A}+2^{B-C}}\times 100,$$

or percentage of HIV-1 genomic RNA relative to a total of three RNAs


$$\frac{1}{1+2^{C-A}+2^{C-B}}\times 100,$$

where *C* is *C_T_* of HIV-1 genomic RNA.

### HIV-1 transmission from 293T cells to different target cells

HEK 293T cells or HEK 293T cells mixed with Cf2Th-CD4/CCR5 cells at a 1:1 ratio were added to wells of a white, flat bottom 96-well plate (Greiner, USA) in 100 µL of complete DMEM at a concentration of 2 × 10^5^ cells/mL. The plate was incubated overnight and then cells were co-transfected with 10 ng reporter plasmid, 8 ng psPAX2, and 2 ng HIV-1 Env- or VSV-G-expression plasmid. pcDNA3.1 was used as a negative control instead of Env expression plasmids. Cells were transfected in triplicates using the Effectene transfection reagent according to the manufacturer’s instructions (Qiagen). The transfection mixture was adjusted to 50 µL per well using a growth medium before adding to cells. In some experiments, the non-nucleoside reverse transcriptase inhibitor Etravirine (NIH AIDS Reagent Program) was added directly to the transfection mixture. At indicated time points or after 48 h, the supernatants were removed and 1× 50 µL of Glo lysis buffer (Promega, USA) was added to each well and incubated for at least 10 min. Saturated-signal samples were further diluted in Glo lysis buffer. To measure nano luciferase activity, NanoGlo luciferase substrate (Promega, USA) was diluted 1:50 in NanoGlo Buffer, and 30 µL of the reagent was added to 50 µL of lysates. Plates were incubated at room temperature for 3 min before luciferase activity was measured with 3-s integration time using Centro XS^3^ LB 960 luminometer (Berthold Technologies, USA). In some experiments, we used methods that have been previously described (3). Briefly, HEK 293T cells were transfected in a 12-well plate (2 × 10^5^ cells in 1 mL of growth medium) with a total of 200 ng of plasmids at the ratios specified above. After 6 h, cells were trypsinized and split equally into two wells. In one well, cells were mixed with target cells (either Cf2Th-CD4/CCR5 or SupT1.CCR5^+^ cells) at a 1:1 ratio and incubated for 36 h. In the second well, cells were allowed to grow and produce HIV-1 virions in the absence of target cells for 18 h; subsequently, virus-containing supernatants were used to infect target cells that were further incubated for 36 h. At the end of the incubation step, cells were lysed using Glo lysis buffer; cell lysates were transferred to a 96-well plate, and nanoluciferase activity was measured as described above.

### HIV-1 transmission between T cells (T cell lines and primary CD4^+^ T lymphocytes)

10^6^ CEM or activated CD4^+^ T cells were washed once with PBS (in 1.5 mL tubes) and electroporated in buffer R with 3 µg of the reporter plasmid, and 2 µg of one of the packaging plasmids, psPAX2, pCMV-dR8.2, or pNL4ΔEnv, in the presence or absence of 1 µg of Env-expression plasmid. In some experiments, the reporter plasmid was co-transfected with 2 µg pNL4-3 or pNL(AD8). Cells were electroporated using Neon Transfection System (Invitrogen, USA), 100 µL tips, and either 1,230 V, 40 ms × 1 pulse for CEM cells, or 1,600 V, 10 ms × 3 pulses for CD4^+^ T cells. Electroporated cells were washed once in 1 mL of culture medium to remove plasmid DNA and then mixed with 5 × 10^5^ SupT1.CCR5 cells; CD4^+^ T lymphocytes were mixed with 5 × 10^5^ autologous CD4^+^ T lymphocytes in a final volume of 1.5 mL growth medium in a 12-well plate for 72 h. Fifty U/mL recombinant human IL-2 was added to the cultures of primary CD4^+^ T cells. To measure Nluc activity, cells were centrifuged (in 1.5 mL tubes) at 1,000 × *g* for 3 min and lysed in 50 µL of Glo lysis buffer. Cell lysates were transferred to a white 96-well plate, and luciferase activity was measured using NanoGlo reagent as described above for adherent cells.

### Viral neutralization assay during coculture of T cells

Antibody was diluted in RPMI culture medium by threefold serial dilutions as previously described (43); control wells were medium without antibodies. Transfected CEM and target SupT1.CCR5 cells, which were prepared as described above, were mixed thoroughly and 50 µL was added into the 96-well plate with diluted antibody. Plates were incubated at 37°C for 72 h, and cells were lysed by adding 30 µL of NanoGlo reagent and aspirating/dispensing several times. Luciferase activity was measured within 3–10 min after the addition of NanoGlo reagent. The same procedure was performed to measure the neutralization of HIV-1 transmission between primary CD4^+^ T cells.

### Free virus infectivity and neutralization assay using T cell lines

To quantify free HIV-1 infection of viral particles produced by T cells, we infected the target cell with free virus-containing supernatant that was collected from the coculture of cells at the end of the cell-cell transmission assay. After 3-day incubation of the cell-cell transmission assay, 1.5 mL of cells was centrifuged and 0.75 mL of the supernatant containing free viruses was added to 5 × 10^5^ SupT1.CCR5 target cells that were resuspended in 0.75 mL fresh medium. Three days later (day 6 of the experiment), cells were lysed, and the level of free HIV infection was evaluated by measuring Nluc activity. In parallel, cell-cell transmission was measured using the centrifuged cells collected on day 3.

For viral neutralization assay in a 96-well format, we transfected 10^6^ CEM-only cells (1 mL), and the cells were grown in a 12-well plate for 72 h. Transfected cells were resuspended and centrifuged at 1,000 × *g* for 3 min (in 1.5 mL tubes). Two-thirds of the supernatant was harvested from the top of the sample and used for the viral neutralization assay. After 72-h incubation, levels of Nluc activity were measured as described above.

### HIV-1 replication in primary CD4^+^ T cells transfected with the reporter vector

10^6^ CEM or primary CD4^+^ T cells were transfected with 5 µg of pUCHR-EF1a-inNluc reporter vector as described above, washed once with medium, and incubated with 300 ng of (p24) HIV-1_NL4-3_ in a final volume of 100 µL for 4 h at 37°C. After a centrifugation step and removal of viral supernatants, cells were washed with 0.5 mL of warm medium and incubated with the indicated target cells in 1.5 mL of culture medium. For the infection of primary CD4^+^ T cells, the medium was supplemented with IL-2. Cell cocultures were incubated in a 12-well plate for the indicated times. Every 2–3 days, cells were suspended and 1 mL of the sample was removed for analysis and replaced with a fresh medium. Cell pellets were lysed in 50 µL of Glo lysis buffer, and Nluc activity was measured as described above; supernatants were used to quantify the level of HIV-1 p24 antigen by ELISA (XpressBio, Frederick, MD, USA).

### Statistical analysis and data presentation

Data are the average + SD of at least three independent experiments. Student’s *t*-test was used to calculate the statistical difference between groups. Dose-response curves of viral neutralization by bnAbs were fitted to the four-parameter logistic equation (44-45) using Prism 7 program (GraphPad, San Diego, CA, USA) after adding the equation to the program; IC_50_ values and the associated s.e. are reported (46–50). The number of experiments and replicates are provided in each figure legend.

## Data Availability

Data are available in the main text or the supplemental materials.

## SUPPLEMENTAL MATERIAL

Supplemental Material mbio.02428-23-s0001.pdf Supplemental figures and tables.

ASM does not own the copyrights to Supplemental Material that may be linked to, or accessed through, an article. The authors have granted ASM a non-exclusive, world-wide license to publish the Supplemental Material files. Please contact the corresponding author directly for reuse.
