# Supplementary material for: Ultrasensitive quantification of HIV-1 cell-to-cell transmission in primary human CD4+ T cells measures viral sensitivity to broadly neutralizing antibodies
Source: mBio. 2023 Dec 8;15(1):e02428-23. doi: 10.1128/mbio.02428-23 (PMC10790777; doi:10.1128/mbio.02428-23)

Ultrasensitive quantification of HIV-1 cell-to-cell transmission in primary human CD4^{+} T cells measures viral sensitivity to broadly neutralizing antibodies

Dmitriy Mazurov and Alon Herschhorn

SUPPLEMENTAL MATERIAL

Glossary

Free virus infection: infection of cells by viruses that have been separated from virus-producing cells.

Cell-to-cell transmission: transfer of HIV-1 from virus-producing cells to target cells through VSs.

Intron-regulated reporter vector: a vector in which reporter protein expression depends on reporter gene splicing to remove an intron.

Luciferase: enzyme catalyzing bioluminescence of substrate without light exposure.

Molecular clone: full-length proviral genome typically cloned into a plasmid vector for production of replication competent virus.

Replication competent virus: a virus that can replicate and spread in cells.

Self-splicing ribozyme: spatially structured intronic RNA catalyzing self-removal without helper proteins.

Single-round infection: viral infection capable of completing only one cycle of replication.

Transduction: viral-mediated transfer of genetic material.

Transmission / infection: assay readout that may include contribution of HIV-1 cell-cell transmission and free HIV-1 infection.

Virological synapses (VSs): a confined environment/structure formed by the interactions of HIV-1 Env on virus-producing cells and CD4 receptor on target cells that facilitates transmission of viruses from virus-producing to target cells.

Table S1. Primers used to assess the minimal length of human γ-intron required for efficient splicing.

Forward primer	Reverse primer	PCR product
GCTCTAGAATTCAGGACAAGTATGGTCATTAAACAGCCTACAGCATACAGG	ATTATGGCCAGTGACTAGTGC	104 bp
GCTCTAGAATTCAGGACAAGTATGGTCATTAAACCTTTAATTCCAGATGGGGGC		251 bp
GCTCTAGAATTCAGGACAAGTATGGTCATTAAAATCTATCTGGAGGCAGGACAA		421 bp

Table S2. RT-qPCR primers used in this study to quantify spliced/unspliced reporter and HIV-1 genomic RNAs in viral particles.

<table>
<tr><th rowspan="2">Intron/ reporter</th><th colspan="2">Unspliced RNA</th><th colspan="2">Spliced RNA</th></tr>
<tr><th>Forward primer</th><th>Reverse primer</th><th>Forward primer</th><th>Reverse primer</th></tr>
<tr><td>in-Luc-mR</td><td rowspan="2">GCCAGTGACTAGTGCTGCAA</td><td rowspan="2">CAGCCTGGCCTCCAGATAAC</td><td>ATTAACGCCCAGCGTTTTCC</td><td>ACACCCGAGGGGGATGATAA</td></tr>
<tr><td>Gb-inNluc</td><td>CGTCGATTACCAGTGTGCCA</td><td>CCCGTATGAAGGTCTGAGCG</td></tr>
<tr><td>Tf-inNluc</td><td>TCGCCGCTCAGACCTTCATA</td><td>GAGTTAGCTAGGCCACCCCA</td><td>CATTTTCACCGCTCAGGACAA</td><td>ACAGACAGCCGGCTACAAC</td></tr>
<tr><td>Rb-inNluc</td><td>GGGTCAACAGCCGTTCAGTA</td><td>GACTTAGGACTTGGCTGCGT</td><td>CTTCAGCCCATTTTCACCGC</td><td>GACAGACCGCTGGCTACAA</td></tr>
<tr><td>HIV-1 genomic RNA</td><td colspan="2">Forward primer: GACATCAAGCAGCCATGCAAAT</td><td colspan="2">Reverse primer: TCTGGCCTGGTGCAATAGG</td></tr>
</table>

Table S3. Average readout (relative light units) of HIV-1 cell-to-cell transmission measured in a 96-well format in the presence and absence of bnAbs.

bnAb	CEM-SupT1 cell system			Primary CD4+ T cells		
	0 μg/ml	1.1 μg/ml	30 μg/ml	0 μg/ml	1.1 μg/ml	30 μg/ml
PGT128	15,294,565	1,640,440	871,231			
PGT121	16,582,305	5,863,471	9,948	284,244	910	889
10-1074	14,807,042	4,730,994	1,613			
3BNC117	13,035,579	6,000,224	1,310			
N6	16,582,305	14,350,783	57,669			
PGT151	12,908,383	14,467,548	1,661,023			
10E8	14,405,412	13,157,143	4,261,206			
VRC01	6,631,937	9,297,742	1,186,488	289,252	302,983	623
2G12	12,377,928	18,545,674	13,374,130			

Table S4. Intron-regulated retroviral reporter vectors[1].

Plasmid ID	Virus	Reporter gene	Intron	Reporter-intron size (bps)	Splicing efficiency (%)	Advantages	Disadvantages	Reference
pUCHR-inLuc	HIV-1	*firefly luciferase*	human γ-globin gene (Full-length intron; *Gb-in*)	2,527	4.8	initial vectors	low sensitivity	(12)
pCRU5HT1-inLuc	HTLV-1				2.6			
pUCHR-inYFP	HIV-1	*yellow FP*		1,593	-			
pCRU5HT1-inYFP	HTLV-1				-			
inGluc-MLV-DERSE	MLV	*gaussia luciferase*		1,432	-	sensitive secreted reporter	high background	(32)
pUCHR-in*G*Luc	HIV-1				-			(3)
pUCHR-inGFPt	HIV-1	*green FP turbo*	shRNA-modified γ-globin intron (*shGb-in*)	1,897	72	efficient splicing and ability to detect single infected cells	low sensitivity	(13)
pCRU5-inGFPt	HTLV-1				81			
pUCHR-in-mCherry	HIV-1	*red FP mCherry*		1,909	80			
pCRU5-in-mCherry	HTLV-1				47			
pCRU5-inLuc-mR	HTLV-1	*firefly luciferase*		2,851	6.1			
pUCHR-inLuc-mR	HIV-1				13-15			
pUCHR-shGb-inNluc		*nano luciferase (Nluc)*	short *shGb-in*[2]	1,309	>99	efficient splicing high sensitivity		this study
pUCHR-EF1a-inNluc								
pTwist-shGb-inNluc					98.9	efficient splicing		
pUCHR-Tf-inNluc			mouse TNFβ	600	41	short introns and vectors	low-moderate splicing efficiency and expression	
pTwist-Tf-inNluc					20			
pUCHR-Rb-inNluc			ribozyme	933	21			
pTwist-Rb-inNluc					19			

[1]Reporter plasmids used in this study are highlighted in light green color. Reporter gene is expressed from CMV promoter in all pUCHR-based plasmids unless otherwise indicated and from the EF1a-HTLV hybrid promoter in all pTwist-based plasmids.
[2] pUCHR-shGb-inNluc contains short shGb intron but plasmids with different lengths of shGb intron were generated in this study.

Additional plasmids used in the current study

Plasmid ID	Synonym	Vector	Backbone	Promoter	Features	Source
psPAX2	-	HIV-1 packaging	pBR322	CMV	No expression of HIV-1 accessory proteins Vpu, Nef, Vpr, and Vif	NIH AIDS Reagent Program
pCMV-dR8.2	pCMV-Δ8.2R				All accessory protein genes intact	Addgene
pNL4ΔEnv	HIV-1$_{NL4\Delta Env}$	Env(-) HIV-1$_{NL4}$ molecular clone		LTR	A frameshift mutation introduced in *env* gene to prevent expression	This study
pNL4-3	HIV-1$_{NL4-3}$	HIV-1 $_{NL4}$ molecular clone			CXCR4-tropic	NIH AIDS Reagent Program
pNL(AD8)	HIV-1$_{1NL4-AD8}$				CCR5-tropic	
pSVIIIe7-AD8	LTR-AD8	HIV-1 Env expression plasmids		LTR	CCR5-tropic Env	The laboratory of Joseph Sodroski
pcDNA 3.1-AD8	CMV-AD8		pcDNA3.1	CMV		
pcDNA 3.1-KB9	KB9				Dual tropic Env	
pCMV-VSVG	VSVG				-	
pGL3-CMV	CMV-Fluc	*Fluc* expression	Promega		-	Promega

Figure S1. Sequences used to engineer in-Nluc reporter plasmids containing the *nanoluciferase* gene.

<u>shGb-in-Nluc</u>:

ACGCGttacgccagaatgcgttcgcacagccgccagccggtcactccgttgatggttactcggaacagcagggagccgtcggggttgatcaggcgctcgtcgataatt
ttgttgccgttccacagggtccctgttacagtgatctttttgccgtcgaacacggcgatgccttcatacggccgtccgaaatagtcgatcatgttcggcgtaaccccgtcgat
taccagGTGAGTCCAGGAGATGTTTCAGCACTGTTGCCTTTAGTCTCGAGGCAACTTAGACAACTGAGTATTGATCTGAGCACAGC
AGGGTGTGAGCTGTTTGAAGATACTGGGGTTGGGGGTGAAGAAACTGCAGAGGACTAACTGGGCTGAGACCCAGTGGCAATG
TTTTAGGGCCTAAGGAATGCCTCTGAAAATCTAGAtgtttgaatgaggcttcagtactttacagaatcgttgcctgcacatcttggaaacacttgctggga
ttacttcttcaggttaacccaacagaaggcTCGAGAAGGTATATGCTGTTGACAGTGAGCGAAGGACAAGTATGGTCATTAAATAGTGAAG
CCACAGATGTATTTAATGACCATACTTGTCCTGTGCCTACTGCCTCGgatcccaaggggctactttaggagcaattatcttgtttactaaaactgaat
accttgctatctctttgatacatttttacaaagctgaattaaaatggtataaattaaatcactTCTAGAATTAATTCAGGACAAGTATGGTCATTAAACAG
CCTACAGCATACAGGGTTCATGGTGGCAAGAAGATAACAAGATTTAAATTATGGCCAGTGACTAGTGCTGCAAGAAGAACAACT
ACCTGCATTTAATGGGAAAGCAAAATCTCAGGCTTTGAGGGAAGTTAACATAGGCTTGATTCTGGGTGGAAGCTGGGTGTGTAG
TTATCTGGAGGCCAGGCTGGAGCTCTCAGCTCACTATGGGTTCATCTTTATTGTCTCCTTTCATCTCAACAGtgtgccatagtgcaggatc
accttaaagtgatgatcatccacagggtacaccaccttaaaaatttttcgatctggcccatttggtcgccgctcagaccttcatacgggatgatgacatggatgtcgatct
tcagcccattttcaccgctcaggacaatcctttggatcggagttacggacaccccgagattctgaaacaaactggatactcctccctgttcaaggacttggtccaggttgt
agccggctgtctgtcgccagtccccaacgaaatcttcgagtgtgaagaccatggtgGCTAGC

<u>TF-in-Nluc</u>:

ACGCGttacgccagaatgcgttcgcacagccgccagccggtcactccgttgatggttactcggaacagcagggagccgtcggggttgatcaggcgctcgtcgataatt
ttgttgccgttccacagggtccctgttacagtgatctttttgccgtcgaacacggcgatgccttcatacggccgtccgaaatagtcgatcatgttcggcgtaaccccgtcgat
taccagtgtgccatagtgcaggatcaccttaaagtgatgatcatccacagggtacaccaccttaaaaatttttcgatctggcccatttggtcgccgctcagaccttcatac
gggatgatgacatggatgtcgatcttcagcccattttcaccgctcagGTGAGGCAGCAAGAGATCTGGGGGTGCTGGGGTGGCCTAGCTAACTC
AGAGTCCTAGAGTCCTCTCCACTCTCTTCTGTCCCAGgacaattcgttggatcggagttacggacaccccgagattctgaaacaaactggacacacctcc
ctgttcaaggacttggtccaggttgtagccggctgtctgtcgccagtccccaacgaaatcttcgagtgtgaagaccatggtgGCTAGC

<u>Rb-in-Nluc</u>:

ACGCGttacgccagaatgcgttcgcacagccgccagccggtcactccgttgatggttactcggaacagcagggagccgtcggggttgatcaggcgctcgtcgataatt
ttgttgccgttccacagggtccctgttacagtgatctttttgccgtcgaacacggcgatgccttcatacggccgtccgaaatagtcgatcatgttcggcgtaaccccgtcgat
taccagtgtgccatagtgcaggatcaccttaaagtgatgatcatccacagggtacaccaccttaaaaatttttcgatctggcccatttggtcgccgctcagaccttcatac
gggatgatgacatggatgtcgatcttcagcccattttcaccgctcaggacaatcctttggatcggagttacggacaccccgagattctgaaacaaactggacacacctcc
ctgttcaaggacttggtccaggttgtagccagcggtAAATAGCAATGGGGTACCACCTTTGCCGCTAAAAGTTATCAGGCATGCACCTGGTAG
CTAGTCTTTAAACCAATAGATTGCATCGGTTTAAAAGGCAAGACCGTCAAATTGCGGGAAAGGGGTCAACAGCCGTTCAGTACC
AAGTCTCAGGGGAAACTTTGAGATGGCCTTGCAAAGGGTATGGTAATAAGCTGACGGACATGGTCCTAACCACGCAGCCAAGT
CCTAAGTCAACAGATCTTCTGTTGATATGGATGCAGTTCACAGACTAAATGTCGGTCGGGGAAGATGTATTCTTCTCATAAGATA
TAGTCGGACCTCTCCTTAATGGGAGCTAGCGGATGAAGTGATGCAACACTGGAGCCGCTGGGAACTAATTTGTATGCGAAAGTA
TATTGATTAGTTTTGGAGTACTCGctgtcgccagtccccaacgaaatcttcgagtgtgaagaccatggtggCCTAGGTCTAGA

EF1a promoter and poly A signal for cloning into pTwist vector

TTCGAACACACAAAAAACCAACACACAGATGTAATGAAAATAAAGATATTTTATTACGCGtta…*inNluc*……catggtggcTAGCGTA
GGCGCCGGTCACAGCTTGGATCTGTAACGGCGCAGAACAGAAAACGAAACAAAGACGTAGAGTTGAGCAAGCAGGGTCAGGC
AAAGCGTGGAGAGCCGGCTGAGTCTAGGTAGGCTCCAAGGGAGCGCCGGACAAAGGCCCGGTCTCGACCTGAGCTTTAAACTT
ACCTAGACGGCGGACGCAGTTCAGGAGGCACCACAGGCGGGAGGCGGCAGAACGCGACTCAACCGGCGTGGATGGCGGCCTC
AGGTAGGGCGGCGGGCGCGTGAAGGAGAGATGCGAGCCCCTCGAAGCTTCAGCTGTGTTCTGGCGGCAAACCCGTTGCGAAA
AAGAACGTTCACGGCGACTACTGCACTTATATACGGTTCTCCCCCACCCTCGGGAAAAAGGCGGAGCCAGTACACGACATCACTT
TCCCAGTTTACCCCGCGCCACCTTCTCTAGGCACCCGTTCAATTGCCGACCCCTCCCCCCAACTTCTCGGGGACTGTGGGCGATGT
GCGCTCTGCCCACTGACGGGCACCGGAGCGATCGCAGATCCCTTGTCGAC

cloning sites

unpaired nucleotides determined by *mfold*

complementary nucleotides in exon (Nluc) and *Tetrahymena* intron participating in ribozyme stem-loop structure formation

a/c/g/t – reversed Nluc gene

A/C/T/G – intron

a/c/g/t – miR30

A/C/G/T – anti-intronic shRNA

a/c/g/t – mutated nucleotides in Nluc

A/C/G/T – reversed β-globin polyA

A/C/G/T – reversed EF1a-HTLV hybrid promoter

Figure S2. Map of the best pUCHR-EF1a-inNluc vector containing shGb-in.

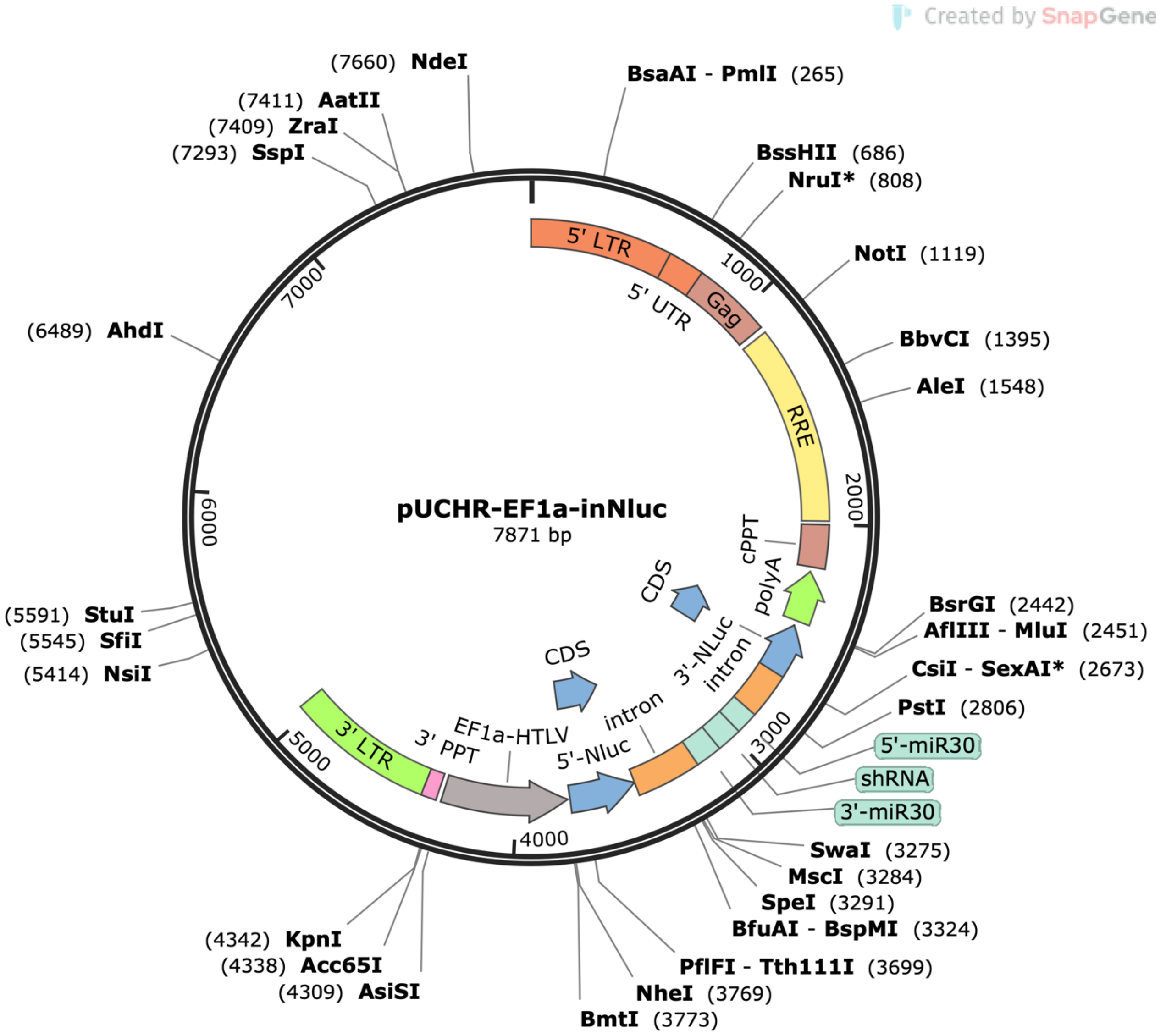

Supplement: Supplemental Material — Supplemental figures and tables. [file mbio.02428-23-s0001.pdf]
